# Bioactive Peptides as Potential Nutraceuticals for Diabetes Therapy: A Comprehensive Review

**DOI:** 10.3390/ijms22169059

**Published:** 2021-08-22

**Authors:** Priya Antony, Ranjit Vijayan

**Affiliations:** Department of Biology, College of Science, United Arab Emirates University, Al Ain P.O. Box 15551, United Arab Emirates; 201990021@uaeu.ac.ae

**Keywords:** diabetes, bioactive peptides, food peptides, α-amylase, α-glucosidase, dipeptidyl peptidase IV

## Abstract

Diabetes mellitus is a major public health concern associated with high mortality and reduced life expectancy. The alarming rise in the prevalence of diabetes is linked to several factors including sedentary lifestyle and unhealthy diet. Nutritional intervention and increased physical activity could significantly contribute to bringing this under control. Food-derived bioactive peptides and protein hydrolysates have been associated with a number health benefits. Several peptides with antidiabetic potential have been identified that could decrease blood glucose level, improve insulin uptake and inhibit key enzymes involved in the development and progression of diabetes. Dietary proteins, from a wide range of food, are rich sources of antidiabetic peptides. Thus, there are a number of benefits in studying peptides obtained from food sources to develop nutraceuticals. A deeper understanding of the underlying molecular mechanisms of these peptides will assist in the development of new peptide-based therapeutics. Despite this, a comprehensive analysis of the antidiabetic properties of bioactive peptides derived from various food sources is still lacking. Here, we review the recent literature on food-derived bioactive peptides possessing antidiabetic activity. The focus is on the effectiveness of these peptides as evidenced by in vitro and in vivo studies. Finally, we discuss future prospects of peptide-based drugs for the treatment of diabetes.

## 1. Introduction

Diabetes mellitus (DM) is a chronic metabolic disorder characterized by persistent hyperglycaemia accompanied by an array of metabolic dysfunctions. It is one of the largest global public health concerns causing high mortality and reduced life expectancy [1]. The three prevalent forms of this disorder are type 1 diabetes mellitus (T1DM), type 2 diabetes mellitus (T2DM) and gestational diabetes mellitus (GDM). Out of these, T2DM accounts for around 90% of the reported cases, 5–7% accounts for T1DM and 2–3% for GDM. T1DM involves autoimmune destruction of pancreatic β-cell that eventually leads to insulin deficiency. The progressive loss of β-cell insulin secretion along with insulin resistance accounts for T2DM. GDM is diagnosed during the second or third trimester of pregnancy [2]. Globally, the incidence of diabetes is rising at an alarming rate. The International Diabetes Federation (IDF) has estimated the global prevalence of diabetes in 2019 to be 9.3% (463 million people), with a projected increase to 10.2% (578 million) by 2030 and 10.9% (700 million) by 2045 [3]. Individuals with T2DM carry a high risk of developing other conditions such as cardiovascular disease, hypertension, stroke, chronic liver disease, chronic kidney disease and cancer [4]. The number of diabetes-related deaths between 2000 and 2019 has risen by almost 70%. According to the World Health Organisation (WHO), diabetes is now on the list of the top 10 causes of death in adults and imposes a heavy burden on public health systems [5]. This rising prevalence of diabetes is linked to several factors such as sedentary lifestyle, ageing, increased urbanisation, unhealthy diet and ubiquitous increase in body-mass index.

Various strategies have been employed to control and manage diabetes including medication, healthy diet, regular physical activity and changing lifestyle. The vast majority of widely used antidiabetic medications mainly focus on stimulating the release of insulin from the pancreas or improving insulin-stimulated glucose uptake [6]. The major classes of conventional antidiabetic drugs include sulfonylureas, biguanides, α-glucosidase inhibitors, peroxisome proliferator-activated receptor-γ (PPARγ) agonists, dipeptidyl peptidase IV (DPP IV) inhibitors, and sodium-glucose co-transporter-2 (SGLT2) inhibitors [7]. These treatment strategies are generally cost-effective. However, long-term use of these drugs could lead to severe complications including hypoglycaemia, vomiting, bloating, potential weight gain, oedema, and problems in the cardiovascular and central nervous systems [8,9]. Nearly half a billion people around the world are living with diabetes. Left unchecked, this situation will inevitably worsen. Hence, there is an urgent need for identifying, developing and implementing safer alternatives to tackle this disorder [3].

Since diabetes is closely linked with lifestyle, not surprisingly, nutritional intervention and increased physical activity could play a vital role in attenuating the problems related to diabetes. Muscular contractions induced by the physical exercises stimulate insulin-independent muscle blood glucose transport and also improves insulin sensitivity [10]. A large body of evidence from clinical trials and observational studies have emphasized the paramount significance of food and dietary patterns in the prevention and management of diabetes [11,12]. Nutraceuticals or functional foods have received significant attention recently due to their multifunctional health benefits.

Cryptides or bioactive peptides are defined as peptide fragments that are generated during proteolytic cleavage or maturation of functional proteins. These fragments may exhibit similar or completely unrelated biological properties in comparison to the parent protein [13]. Food-derived bioactive peptides are short sequences of 2–50 amino acids that are embedded within the primary structure of dietary proteins [14]. Normally, these peptide sequences are inactive in the precursor protein and are activated during food processing or enzymatic hydrolysis [8]. The precise function of these peptides depends on their amino acid composition, sequence and length [15]. A growing body of scientific evidence now indicates that peptides regulate a number of physiological processes and could act as antidiabetic, antihypertensive, antimicrobial, opioid, antioxidant, anticancer, and immunomodulatory agents [16,17,18]. To date, more than 80 peptide drugs have reached the market for various disorders including diabetes, cancer, osteoporosis, and multiple sclerosis. Additionally, several are in clinical and preclinical trials [19]. In this context, a large number of bioactive peptides possessing antidiabetic activity have been derived from various food sources including milk, egg, fish, pulses, legumes, and cereals [18,20]. These peptides regulate blood glucose levels by inhibiting major enzymes such as α-amylase, α-glucosidase, and DPP IV as well as acting as an agonist of glucagon-like peptide 1 (GLP-1) [21].

The focus of this review is to comprehensively evaluate the current knowledge of food-derived bioactive peptides possessing antidiabetic activity. Specific attention has been paid to identify the effectiveness of these peptides as evident from in vitro and in vivo studies. Furthermore, this review discusses the challenges involved in the development of and future prospects of peptide-based drugs for the treatment of diabetes.

## 2. Functional Foods and Bioactive Peptides

Functional foods refer to foods that provide potential health benefits beyond serving as a nutritional source. Epidemiological studies have highlighted the importance of dietary foods in managing and reducing the complications associated with diabetes [22]. Regular intake of these, including whole foods, enriched foods, and probiotics, could help with glycaemic control, pancreatic β-cell function, insulin secretion, regulation of lipid metabolism, and weight management.

As endogenous signalling molecules for several intracellular processes, peptides represent an excellent starting point for designing novel drug molecules. Furthermore, peptides are highly selective molecules with an exquisite potency and safety profiles in humans. The generally favourable pharmacological profile of peptides makes them a unique class of pharmaceutical compounds [23]. This has led to a significant growth in the utilization of bioactive peptides for managing chronic disorders in recent years. Functional foods are packed with phytochemicals, vitamins, minerals, fibre, antioxidants, fatty acids, and bioactive peptides. While many of these constituents have been widely studied, food-derived bioactive peptides have come into focus due to their nutritional capabilities and health benefits. These peptides are usually encrypted within the sequence of their parent protein and are released during maturation, or chemical, microbial, or enzymatic hydrolysis [13]. In a large number of instances, bioactive peptides liberated from the parent protein were shown to possess bioactivity that may or may not be similar to the parent protein. A myriad of bioactive peptides with multiple health benefits have been identified from various food sources. The most widely explored food sources from animals include milk, egg, and meat proteins. Bioactive peptides have also been isolated from vegetable proteins such as pulses, lentils, wheat, rice, oat, and flaxseeds. Proteins from marine organisms such as fish, tuna, salmon, squid, oyster, and crabs are also excellent sources of bioactive peptides [24,25,26]. Bioactive peptides from food sources are mainly produced by hydrolysis using digestive enzymes such as trypsin and pepsin, microbial fermentation, and food processing by pickling, canning, drying, and smoking. By far the most effective methods employed for producing peptides from food sources includes microbial fermentation and enzymatic digestion [27]. Fermentation involves the culturing of proteolytic microbes on a protein that helps in yielding shorter peptides. It represents a cost-effective method for producing natural bioactive peptides, especially in the dairy industry. Some preferred starter cultures used for the fermentation process include yeast, fungi, and microbes from the *Lactobacillus* genus [28]. Apart from obtaining bioactive peptides, studies have shown that fermentation improves the functional and nutritional quality of food, which in turn provides numerous health benefits [29]. Proteolytic enzyme digestion of food proteins is another efficient approach to release potentially active peptides. Some commonly used enzymes used for hydrolysis include pepsin, trypsin, chymotrypsin, alcalase, and flavourzyme. These enzymes hydrolyse peptide bonds and release peptides of different sizes and properties. Compared to microbial fermentation, enzymatic hydrolysis produces bioactive peptides in a short time with a high scalability. In both methods, bioactivity, size, and bioavailability of the released peptides largely depend on time, degree of hydrolysis, processing conditions, enzyme-substrate ratio, and pre-treatment of proteins [30]. Peptide fragments released from hydrolysed food proteins typically contain several biologically active peptides that could positively alter physiological function and reduce disease risk. They could also impart beneficial effects on endocrine, cardiovascular, digestive, nervous, and immune systems [31].

## 3. Current Diabetes Targets

Several therapeutic approaches are currently being employed for managing blood sugar levels and increasing insulin sensitivity. Carbohydrate metabolism is managed by a complex interplay of organs, glands, and secretions [32]. Blood sugar levels are largely regulated by the action of the two peptide hormones insulin and glucagon [33]. The use of insulin from animal pancreas began in the 1920s and is often regarded as a pioneering step in peptide therapeutics that revolutionised the treatment of T1DM [34].

Since the 1990s, α-glucosidase and α-amylase inhibitors have been considered first-line drugs for the treatment and management of T2DM [35,36]. As these digestive enzymes are involved in the catabolism of complex carbohydrates into glucose, inhibiting these enzymes is considered an effective strategy for decreasing blood sugar levels [37]. Another widely used treatment option is the inhibition of DPP IV and the activation of GLP-1 receptors using GLP-1 analogues and incretin mimetics [37,38]. GLP-1 is an important incretin hormone that helps in managing blood glucose levels by stimulating insulin release, inhibiting glucagon production, and slowing gastric emptying. However, this endogenous hormone is rapidly degraded and inactivated by DPP IV [39]. Given the significance of these molecules in managing diabetes, synthetic DPP IV inhibitors and GLP-1 receptor agonists were developed to regulate these pathways [40]. Other oral hypoglycaemic drugs include SGLT2 inhibitors, which reduce blood sugar levels by preventing reabsorption of renal glucose, and insulin sensitizers such as biguanides that improve insulin sensitivity, increase glucose uptake and prevent the production of glucose in the liver [41]. Insulin secretagogues, which stimulate insulin secretion, and thiazolidinediones (TZD), which reduce the insulin resistance in adipocytes, liver and muscles, are other treatment options used for managing diabetes [42]. Even though the number of treatment options has significantly grown over the past few decades, none of these drugs are capable of stopping the progressive decline of β-cell function [43]. The challenges and limitations of existing therapeutics have prompted the search for developing alternative or complementary drugs that are effective and have fewer, lower, or no side effects.

## 4. Peptide Drugs and Diabetes

In spite of the development of the therapeutic use of insulin in the early 1920s, peptide therapeutics have evolved at a slower pace over time. Even though peptides act as key biological modulators with remarkable potency, low toxicity and selectivity, limitations such as low oral bioavailability, stability, short circulation time, and cost have limited the interest of pharmaceutical companies in developing peptide-based drugs. The introduction of recombinant technology has provided a reliable and efficient option for the production of peptide drugs. The development of human insulin using recombinant technology (1982), as well as other synthetic hormones, have firmly established the market for peptide-based drugs [44]. Along with this, the discovery of natural peptide leads, from various sources including plants, animals, and microorganisms, has opened up several avenues to pursue the development of more potent antidiabetic drugs. Moreover, advancements in peptide synthesis, formulation, and drug delivery could encourage the industry to invest in peptide-based drug research and development.

In recent years, peptide drugs have received significant attention as potential leads due to their beneficial properties. Compared to small molecule drugs, peptides could be more potent and tissue-specific with negligible side effects. In the last few years (2015–2019), the US Food and Drug Administration (FDA) has approved 15 peptide drugs that accounted for 7% of the total drugs approved during this period [45]. Recent advancements in peptide screening and computational biology approaches have assisted in the development of novel peptide drugs. Peptide drugs that are currently used for the treatment of diabetes are derived from a number of different sources. One of the most promising GLP-1 receptor agonists currently used for the treatment of diabetes is exedin-4. It was derived from the venom of *Heloderma suspectum* (Gila monster) and approved by the FDA in 2005. A more potent synthetic version, exenatide, was also introduced [46]. Despite various oral medications available for T2DM, the market for injectable exedin-4 has continued to grow since its approval. Following this, various other peptide drugs such as liraglutide and semaglutide with increased stability and improved half-life were approved by the FDA [47]. Protein hydrolysates and peptides from food sources have already been commercialized and are being used as health promoting agents. For instance, Nutripeptin^TM^ is a marine bioactive peptide obtained from the proteolytic hydrolysis of cod fish fillets. It lowers the glycaemic index of foods, which in turn reduces the risk of T2DM. The growing interest of bioactive peptides as a safer and promising antidiabetic agent has spearheaded the search for finding more peptide-based lead molecules for the treatment of diabetes.

## 5. Antidiabetic Peptides from Food Sources

**Figure 1:**
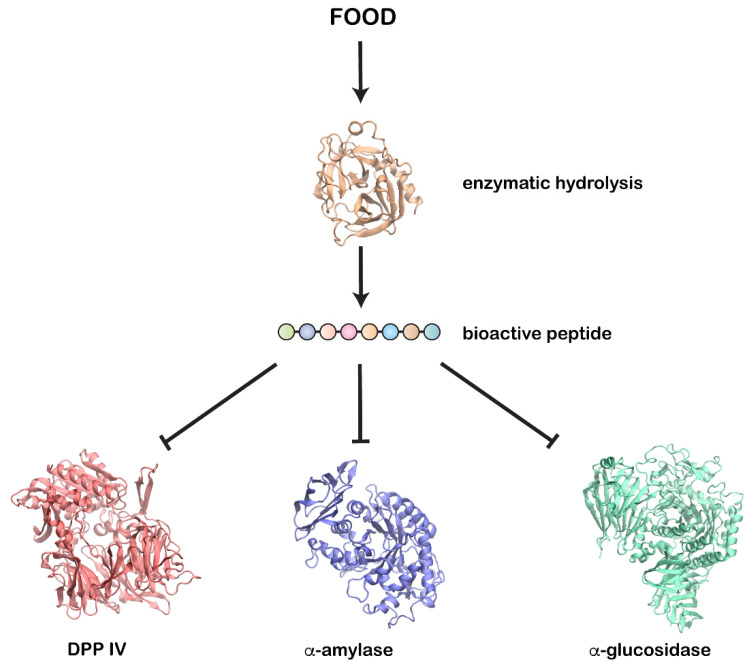
Enzymatic hydrolysis of food proteins produces bioactive peptides that could inhibit key enzymes involved in diabetes—DPP IV, α-amylase, and α-glucosidase.

Bioactive peptides from several food sources have been reported to show antidiabetic activity by inhibiting carbohydrate digesting enzymes (α-amylase and α-glucosidase) and DPP IV (Figure 1), enhancing pancreatic insulin secretion, controlling satiety, and reducing glucose absorption from the gut [18].

### 5.1. Animal Products

#### 5.1.1. Dairy Foods

Dairy products such as milk, cheese, yoghurt, and other cultured dairy foods are dominant sources of bioactive peptides. Milk proteins are a versatile source of bioactive peptides as they possess numerous health benefits including antidiabetic, antioxidant, antihypertensive, antimicrobial, and immunomodulatory properties [48]. Animal milk is a nutrient-rich fluid secreted by the epithelial cells of the mammary glands. The two major proteins present in milk are casein and whey. Both of these have been identified as rich sources of bioactive peptides [49]. Since peptides and hydrolysates from milk could act as antidiabetic agents, they have attracted significant interest from the scientific community [50]. Perhaps the most widely consumed animal milk originates from cows. Therefore, antidiabetic potential of cow milk proteins and their hydrolysates has been investigated extensively [51]. Oral administration of cow milk proteins and their hydrolysates on diabetic rats significantly reduced the concentration of blood glucose, total lipids, triglycerides, and cholesterol as well as increased the concentration of globulin and high-density lipoproteins (HDL) [52]. Administration of donkey milk has been demonstrated to improve the viability of damaged pancreatic β-cells in mouse insulinoma beta-pancreatic (MIN6) cells. Furthermore, diabetic rats fed with donkey milk powder for four weeks showed a significant decrease in blood glucose levels, increased insulin sensitivity and improved insulin resistance. It has been suggested that donkey milk could be used as an antidiabetic agent as it downregulated phosphoenolpyruvate carboxykinase 1 (PCK1) and glucose-6-phosphatase (G6Pase), enzymes that are involved in hepatic gluconeogenesis [53]. The peptides SDIPNPIGSE, NPWDQVKR, SLSSSEESITH, and QEPVLGPVRGPFP isolated from goat milk casein hydrolysates showed a significant improvement in glucose metabolism in insulin-resistant HEPG-2 cells. Interestingly, some of these casein-derived sequences are also conserved in sheep, buffalo, and cow. However, more work needs to be done to establish if these peptides are indeed released and are functional. Goat milk was also observed to decrease the mRNA level of PCK1 and G6Pase and in turn improved glycogen concentration. Thus, these findings suggest that peptides from goat milk could ameliorate insulin resistance and manage type 2 diabetes [54]. Zhang et al. identified novel DPP IV inhibitory peptides from trypsin- and chymotrypsin-treated goat milk casein. Out of the five new identified peptides (MHQPPQPL, SPTVMFPPQSVL, VMFPPQSVL, INNQFLPYPY and AWPQYL), the peptide INNQFLPYPY derived from the κ-casein displayed highest inhibitory activity with an IC_50_ value of 40.08 μM [55]. Studies have also identified camel milk peptides that could potentially inhibit key enzymes like DPP IV and amylase [56]. Trypsin-digested camel milk proteins were shown to produce the peptides FLQY, FQLGASPY, ILDKEGIDY, ILELA, LLQLEAIR, LPVP, LQALHQGQIV, MPVQA, and SPVVPF that could inhibit DPP IV [57]. Thus, a range of studies involving widely consumed animal milk have underscored the importance of milk proteins and hydrolysates in managing diabetes (Table 1).

Due to high nutritional value and protein content, milk-derived food products such as yoghurt and cheese are often consumed as part of a balanced diet. In recent years, the health benefits of yoghurt have been studied extensively and it has been shown to reduce the risk of several metabolic disorders. Regular intake of yoghurt could modulate glucose metabolism and reduce the risk associated with diabetes [58]. Microbial fermentation that produces yoghurt generates bioactive peptides that have antidiabetic potential. Addition of aqueous berry extracts (salal berry and blackcurrant pomace) to yoghurt improved blood glucose regulation by the inhibition of α-amylase, α-glucosidase and DPP IV enzymes. Using liquid chromatography coupled with mass spectrometry (LC/MS), nearly 486 peptides were identified from yoghurt possessing diverse bioactivities [59]. Water-soluble extracts of gouda-type cheese has been shown to exhibit DPP IV inhibitory activity. Among several peptides that were identified, LPQNIPPL exhibited the highest DPP IV inhibitory activity. Interestingly, oral administration of this peptide to female rats reduced blood glucose concentration. Furthermore, the concentration of LPQNIPPL was found to increase 4.3-fold in cheese ripened for a year [60].

**Table 1:** Antidiabetic action of peptides and protein hydrolysates identified from animal food sources.

Source	Enzyme(s) Used	Substrate	Protein Hydrolysate	Peptide(s) Identified	Mechanism of Action	IC_50_	Reference
Cow milk	Trypsin	Milk protein	Milk protein hydrolysates		Reduced blood plasma glucose level in vivo		[52]
Donkey milk	Pepsin and pancreatin		Milk protein hydrolysates		Improved damaged β-cells viability in vitro, reduced blood glucose level and improved insulin resistance in vivo		[53]
Goat milk	Flavourzyme	Casein	Casein hydrolysate	SDIPNPIGSE	Ameliorated insulin resistance in HepG2 cells		[54]
				NPWDQVKR			
				SLSSSEESITH			
				QEPVLGPVRGPFP			
Goat milk	Trypsin and chymotrypsin	Casein	Casein hydrolysate	MHQPPQPL	Inhibited DPP IV in vitro	350.41 ± 4.1 μM	[55]
				SPTVMFPPQSVL		676.31 ± 12.6 μM	
				VMFPPQSVL		Nd	
				INNQFLPYPY		40.08 ± 5.0 μM	
				AWPQYL		Nd	
Camel milk	Alcalase, bromelain, and papain	Milk proteins	Milk protein hydrolysates	MPSKPPLL	Inhibited pancreatic α-amylase in vitro		[56]
				KDLWDDFKGL			
Camel milk	Trypsin	Milk proteins	Milk protein hydrolysates	FLQY	Inhibited DPP IV in vitro	>1000 μM	[57]
				FQLGASPY		>1000 μM	
				ILDKEGIDY		347.8 ± 42.8 μM	
				ILELA		721.1 ± 46.3 μM	
				LLQLEAIR		177.8 ± 22.2 μM	
				LPVP		87.0 ± 3.2 μM	
				LQALHQGQIV		>1000 μM	
				MPVQA		93.3 ± 8.0 μM	
				SPVVPF		214.1 ± 16.7 μM	
Gouda cheese	Microbial fermentation	Water-soluble extracts (WSEs)		LPQNIPPL	Reduced glucose level in vivo and inhibited DPP IV in vivo and in vitro	46 μM	[60]
				LPQ		82 μM	
				VPITPTL		110 μM	
				VPITPT		130 μM	
Egg	Alcalase	Egg white protein	Egg white protein hydrolysates	RVPSLM	Inhibited α-glucosidase in vitro	23.07 μmol/L	[61]
				TPSPR		40.02 μmol/L	
				DLQGK		>150.0 μmol/L	
				AGLAPY		>150.0 μmol/L	
				RVPSL		>150.0 μmol/L	
				DHPFLF		>150.0 μmol/L	
				HAEIN		>150.0 μmol/L	
				QIGLF		>150.0 μmol/L	
Egg	Pepsin	Egg yolk protein	Egg yolk protein hydrolysates	YINQMPQKSRE,	Inhibited DPP IV in vitro	222.8 μg/mL	[62]
				VTGRFAGHPAAQ	Inhibited α-glucosidase in vitro	365.4 μg/mL	
Turtle egg	Trypsin, pepsin, α-chymotrypsin, thermolysin, and GI enzyme	Egg yolk protein	Egg yolk protein hydrolysates	LPSW	Inhibited DPP IV in vitro and in silico	289.2 ± 11.85 μM	[63]
				WLQL		269.7 ± 15.91 μM	
				LPLF		463.6 ± 5.52 μM	
				VPGLAL		>2000	
				LVGLPL		432.5 ± 40.31 μM	
Chicken egg	Pepsin and trypsin	Myosin and lysozyme		ADF	Inhibited DPP IV in vitro and in silico	16.83 mM	[64]
				MIR		4.86 mM	
				FGR		46.2 mM	
Cooked meat (pork, beef, chicken, and turkey)	Amylase, pepsin and trypsin	Meat proteins	Meat protein hydrolysates	IPI	Inhibited DPP IV in vitro	3.5 μmol/L	[65]
				WL		44 μmol/L	
				LPL		241 μmol/L	
				WI		89 μmol/L	
				FL		400 μmol/L	
				LW		993 μmol/L	
Cooked meat (Pork, chicken and turkey)				VL		74 μmol/L	
				WM		243 μmol/L	
				ML		91 μmol/L	
Cooked meat (Pork and beef)				IP		150 μmol/L	
				LP		712 μmol/L	
				AL		882 μmol/L	
Cooked meat (Chicken and turkey)				FP		363 μmol/L	
Cooked meat (Pork)				IPM		70 μmol/L	
Dry cured ham				KA	Inhibited DPP IV in vitro	6.27 ± 0.59 mM	[66]
				AA		9.40 ± 0.10 mM	
				GP		9.69 ± 0.49 mM	
				PL		>10 mM	
				AAATP		6.47 ± 0.20 mM	
				AAAAG		8.13 ± 0.48 mM	
				ALGGA		>10 mM	
				LVSGM		>10 mM	
Chicken feet	Neutrase and protamex	Feet protein	Feet protein hydrolysates		Stimulated GLP-1 release in vivo and inhibited DPP IV in vitro	4.42 ± 0.1 μL	[67]
Chicken by product	Flavourzyme and corolase	Mechanical chicken deboning residue	Protein hydrolysates	TL	Promoted glucose uptake and inhibited DPP IV in vitro		[68]
				HT			
				LA			
				LADVEVDLL			
				LL			
				ETGKGEDGE			
				FL			
				LFFSMLLML			
				LF			

Nd—Not determined.

#### 5.1.2. Eggs

As part of a balanced diet, eggs are generally recommended as a good source of protein and nutrients. Beyond serving as an excellent nutrient-dense food source, egg proteins harbour bioactive peptides that perform diverse biological activities [69]. Proteolysis of egg proteins using pepsin and alcalase have been reported to produce bioactive peptides with antidiabetic property. Hydrolysis of egg white protein using alcalase produced eight peptides. Among these, RVPSLM and TPSPR exhibited the highest α-glucosidase inhibitory activity [61]. Peptides YINQMPQKSRE and VTGRFAGHPAAQ from chicken egg yolk pepsin hydrolysate was shown to inhibit both α-glucosidase and DPP IV. Notably, compared to peptides produced by alcalase digestion, these peptides exhibited better antidiabetic activity [62]. Five novel DPP IV inhibitory peptides LPSW, WLQL, LPLF, VPGLAL, and LVGLPL, were identified from the egg yolk of the soft-shelled turtle. Out of these, LPSW competitively inhibited DPP IV with an IC_50_ of 269.7 ± 15.91 µM [63].

Often, in silico virtual screening is employed to identify novel peptides since this method is cost-effective, saves time and is dependable. In silico digestion of chicken egg proteins by pepsin and trypsin has been used to identify antidiabetic and antihypertensive peptides. The peptides ADF, MIR, and FGR obtained from myosin and lysozyme have been shown to be able to inhibit DPP IV [64] (Table 1). Additionally, whole peptidomics and bioinformatics study of chicken egg white has revealed the presence of 43 novel antidiabetic peptides based on homologous sequence motifs identified in other food-derived peptides [70].

#### 5.1.3. Meat Products

Animal meat is widely consumed and serves as a rich dietary source of proteins, minerals, and vitamins. Bioactive peptides generated from the hydrolysis of meat from cattle, sheep, goats, pigs, and poultry have been identified to play a crucial role in various physiological processes [24]. Apart from meat, by-products such as blood, skin and bone could also serve as a source of peptides [71]. In vitro digestion of four types of cooked meat, including pork, beef, chicken, and turkey, using gastrointestinal enzymes producing peptides with antioxidant, angiotensin-converting enzyme (ACE) inhibitory and DPP IV inhibitory properties. Out of these, pork meat was found to be an excellent source of DPP IV inhibitory peptides [65]. Peptide fractions of digested meat yielded 23 DPP IV inhibitory peptides with the highest activity shown by the tripeptide IPI (diprotin A) with an IC_50_ of 3.5 μmol/L [65]. In silico digestion of pork meat proteins using gastrointestinal enzymes identified small dipeptides that could improve glucose absorption and DPP IV inhibition [72]. Processing of meat by drying, curing, ripening, and fermentation are some of the earliest approaches used for the preservation and enhancement of flavour. Such processing often releases encrypted bioactive peptides from parent proteins [71]. Dry-cured ham is an excellent source of bioactive peptides [73]. A regular intake of 80 g of Spanish dry-cured ham has been shown to be protective against cardiovascular disorders and could lower blood glucose levels [74]. Added to this, peptides from dry ham could also act as DPP IV and α-glucosidase inhibitors. Nine peptides were isolated from Spanish dry-cured ham and peptides KA and AAATP exhibited strong DPP IV inhibitory activity, with IC_50_ of 6.27 mM and 6.47 mM [66]. Similarly, 63 peptides were identified from Iberian dry-cured ham. Out of these, eight peptides (GGLGP, LGVGG, AEEEYPDL, EA, PP, VE, PE, AD) were previously reported to be bioactive and these were tested for α-glucosidase inhibitory activity. The results confirmed the multifunctionality of these peptides [75].

Bioactive peptides have also been isolated from poultry products. Protein hydrolysates from chicken feet showed high DPP IV inhibitory capacity with an IC_50_ of 300 μg/mL. Furthermore, glucose tolerance test in diet and age-induced glucose-intolerant Wistar rat models revealed that the peptides improved plasma glucose profile and stimulated GLP-1 release [67]. In another study, peptides isolated from chicken by-product, such as residue from mechanical deboning of chicken, exhibited potential antidiabetic activity by inhibiting DPP IV in vitro and promoting ex vivo cellular glucose uptake. Among the 14 DPP IV inhibitory peptides identified, the highest inhibitory activity was observed in peptides with an N-terminal leucine or isoleucine residue [68] (Table 1).

### 5.2. Fish Products

Fish is one of the healthiest food sources, owing to its low fat and high-quality protein content. It is loaded with minerals and vitamins and is also a great source of omega-3 fatty acids. Including fish as a part of a healthy diet could reduce the incidence of various metabolic disorders. Protein hydrolysates or peptides isolated from various fish species has the potential to significantly improve glucose metabolism (Table 2). Silver carp (*Hypophthalmichthys molitrix* Val.) is one of the major freshwater fish in China. Combining in vitro and in silico analysis, Zhang et al. investigated the DPP IV inhibitory activity of silver carp protein (SCP) hydrolysates. Four peptides—AGPPGPSG, ALAPSTM APGPAGP, and LPIIDI—were identified from the protein hydrolysates fraction that exhibited high DPP IV inhibitory activity. All these peptides were assumed to be good inhibitory agents due to the presence of proline in their sequence. Out of these peptides, LPIIDI and APGPAGP showed high DPP IV-inhibitory activity with an IC_50_ value of 105.44 and 229.14 μM [76]. Protein hydrolysates generated from the enzymatic digestion of silver carp swim bladder protein also exhibited DPP IV inhibitory activity. The protein fraction that exhibited the highest inhibitory action contained 40 different peptides of varying lengths. The most potent peptide WGDEHIPGSPYH inhibited DPP IV in an uncompetitive manner and docking analysis indicated that the peptide bound close to the catalytic site of the enzyme. Furthermore, this peptide and its hydrolysate IPGSPY exhibited strong DPP IV inhibition and promoted insulin secretion in Caco-2 cells [77]. Three peptides, PGVGGPLGPIGPCYE, CAYQWQRPVDRIR, and PACGGFWISGRPG, that produced in vitro DPP IV inhibitory activity with IC_50_ of 116.1, 78.0 and 96.4 μM, respectively, were identified from tuna cooking juice [78]. In vitro and in vivo analysis of blue whiting (*Micromesistius poutassou*) protein hydrolysate was found to inhibit DPP IV and promoted the release of insulin and GLP-1 in pancreatic BRIN-BD11 and GLUTag cells. Similarly, protein hydrolysates from salmon by-products such as skin and trimmings have exhibited DPP IV inhibitory activity and improved insulin and GLP-1 secretion in pancreatic BRIN-BD11 and GLUTag cells [79,80].

### 5.3. Plant Products

A large number of bioactive peptides and protein hydrolysates with potential health benefits have been isolated and identified from plant-based food sources.

#### 5.3.1. Pulses and Legumes

Pulses and legumes represent the cheapest source of essential proteins that are consumed all over the world. Soybeans are a rich source of high-quality proteins including all essential amino acids required for human health. Storage proteins abundantly found in soybean include β-conglycinin and glycinin [81]. Epidemiological studies have suggested that soy consumption could be associated with the alleviation of chronic disorders such as cardiovascular disease, diabetes, obesity, cancer and other immune disorders [81]. Diverse bioactive peptides isolated from soybeans and fermented or processed soybean products such as soymilk, tofu, soy sauce, tempeh, natto and soy paste are gaining interest due to their health benefits [82]. Several studies have also highlighted the importance of soy peptides in the management of diabetes and insulin resistance [83,84]. The pharmacological activity of aglycin, a 37 amino acid long peptide isolated from soybean has been investigated in vivo [85]. Oral administration of the peptide (50 mg/kg) to diabetic mice was shown to effectively control hyperglycaemia by the enhancement of the insulin-signalling pathway and an improvement in glucose uptake in peripheral tissues. Additionally, this natural peptide is highly stable and resistant to gastrointestinal digestive enzymes such as trypsin and pepsin [85]. Pharmacological and animal model studies have been performed using vglycin, another 37 amino acid long peptide isolated from soybean, to determine the effect on the proliferation and restoration of pancreatic β-cells. The results suggest that this peptide could improve glucose tolerance, restore pancreatic function and enhance insulin signalling by activating the insulin receptor (IR)/Akt signalling pathway [86,87]. Long term administration of soymorphin-5 (YPFVV), a soy-derived μ-opioid peptide derived from the β-subunit of β-conglycinin, improved glucose and lipid metabolism in diabetic KKAy mice by activating the adiponectin and peroxisome proliferator-activated receptor α system [88]. In vitro studies using germinated soybean peptides were found to modulate hyperglycaemia by inhibiting DPP IV, salivary α-amylase, and intestinal α-glucosidases enzymes [89]. The inhibitory activity of soy peptide IAVPTGVA and lupin bean peptide LTFPGSAED were investigated by performing DPP IV assays in situ on human intestinal Caco-2 cells and ex vivo on human serum. These peptides inhibited DPP IV with an IC_50_ of 223.2 and 207.5 µM. Furthermore, they displayed inhibitory action on circulating DPP IV with a slighter lower potency when compared to in vitro and in situ studies [90]. These results are in agreement with the inhibition of DPP IV activity in situ in human intestinal Caco-2 cells by soybean protein hydrolysates [91] (Table 3).

Apart from this, fermented soybean products are very popular in Asian countries including Japan, China, Korea, Thailand, and Indonesia. Fermentation results in the release of bioactive peptides by the action of proteolytic enzymes produced by microorganisms [92]. Interestingly, compared to unfermented soybeans, fermented soybean products have been shown to exhibit better antidiabetic properties in animal and human studies [93]. Fermented soybean fed to streptozotocin (STZ)-induced diabetic rats inhibited α-amylase and intestinal α-glucosidase. Fermented soymilk is an excellent source of bioactive peptides and fermentation of soymilk by kefir, which are colonies of microorganisms primarily involving lactic acid bacteria, was observed to inhibit α-amylase [94]. Clinical studies have also established that consumption of fermented soy foods (natto and miso) is associated with lowering the incidence of gestational diabetes [95]. DPP IV inhibitory dipeptides KL and LR have been isolated from water-soluble extract of natto [96].

Bioactive peptides from different varieties of bean have also been found to be beneficial for the treatment of diabetes. Biochemical and in silico analysis have been employed to identify potential bioactive peptides from black bean (*Phaseolus vulgaris*) protein. Molecular docking analysis suggested that the peptides EGLELLLLLLAG, AKSPLF, and FEELN could potentially inhibit DPP IV, TTGGKGGK could inhibit α-glucosidase, and AKSPLF and WEVM could inhibit α-amylase [97]. Using phage display approach 5 novel α-amylase inhibitory peptides, PPHMLP, PLPTGAGF, PPHMGGP, PLPLHMLP, and LSSLEMGSLGALFVCM were identified from pinto beans [98]. The effectiveness of these peptides was further evaluated in AR42J cells. The results demonstrated that these peptides could indeed inhibit α-amylase with LSSLEMGSLGALPVCM exhibiting the best inhibition with an IC_50_ of 0.31 mM [99]. Similarly, peptides and protein hydrolysates from easy-to-cook (ETC) and hard-to-cook (HTC) beans were also shown to lower blood glucose levels by inhibiting α-glucosidase [100]. Bambara bean (*Vigna subterranean*) protein hydrolysates digested using alcalase and thermolysin also exhibited significant DPP IV inhibitory properties [101]. Peptides from cowpea (*Vigna unguiculata*) have been demonstrated to activate the insulin signalling pathway in vivo in rat skeletal muscles. L6 rat skeletal muscles were treated with various doses of cowpea peptides (0.1, 1, 10 and 100 ng) for 20 h or insulin (100 nM) for 30 min. Administration of cowpea peptides induced phosphorylation of Akt (protein kinase B) that eventually activated the insulin signalling cascade [102]. Peptides produced from chickpea (*Cicer arietinum*) using a simulated digestive system and bromelain (enzyme derived from pineapple plant) hydrolysis were compared for their antidiabetic activity. Inhibition assays and molecular docking analysis indicated that the peptides PHPATSGGGL and YVDGSGTPLT had greater affinity for the catalytic site of DPP IV compared to peptides isolated from bromelain hydrolysate [103]. Evidently, protein-rich beans are a good source of bioactive peptides, many of which are yet to be fully explored.

#### 5.3.2. Cereals and Pseudocereals

Since ancient times, cereal grains have been consumed as a staple around the world. Protein hydrolysates and peptides from cereals and pseudocereals have been shown to inhibit enzymes associated with diabetes and could maintain glucose homeostasis by improving insulin sensitivity [104]. Oral administration of dietary corn and wheat-based peptides to nonobese diabetic (NOD) mice have delayed the onset and reduced the incidence of type 1 diabetes by reducing the inflammation in mouse models [105]. Peptide hydrolysates from three cereals—oat, buckwheat and highland barley seeds—have been evaluated for DPP IV inhibitory activity. Hydrolysates of all three cereals strongly inhibited DPP IV activity (highland barley, IC_50_ of 3.91 mg/mL; buckwheat, IC_50_ of 1.98 mg/mL; oat, IC_50_ of 0.99 mg/mL). From the hydrolysates, peptides LQAFEPLR and EFLLAGNNK obtained from oat storage proteins showed the highest degree of DPP IV inhibition in molecular docking and in in vitro assays [106]. Nine protein hydrolysates from wheat gluten exhibited DPP IV inhibitory activity and the most effective hydrolysate was further processed to identify potent peptides. VPL, WL, and WP were subsequently shortlisted using liquid chromatography tandem mass spectrometry (LC-MS/MS) [107] (Table 3).

Quinoa is a pseudocereal with a very high protein and amino acid content compared to other dietary grains. The antidiabetic properties of quinoa protein have been demonstrated [108]. Bioactive peptides from quinoa protein hydrolysates were found to be potent DPP IV inhibitors. Quinoa peptides GEHGSDGNV, IQAEGGLT, and DKKYPK exhibited antidiabetic activity by inhibiting DPP IV, α-amylase, or α-glucosidase [109]. In silico digestion of quinoa globulin protein yielded four promising tripeptides MAF, NMF, HPF, and MCG that exhibited DPP IV and ACE inhibitory activity [110]. Like quinoa, amaranth is another pseudocereal with high nutritional value and nutraceutical properties. Bioactive peptides isolated from enzymatic digestion of amaranth seed proteins were reported to inhibit DPP IV in in vitro, in vivo and in silico studies [111]. Hydrolysates of amaranth grain storage proteins albumin 1, globulin and glutelin hydrolysates (GluH) competitively inhibited DPP IV activity in STZ-induced diabetic mice. GluH was observed to improve glucose tolerance and plasma insulin [111]. Additionally, in vitro simulated gastrointestinal digestion (SGID) of *Amaranthus caudatus* proteins released multifunctional peptides that possessed DPP IV and α-amylase inhibitory properties. This included peptides FLISCLL, SVFDEELS, DFIILE, NRPET, and HVIKPPS that could potentially inhibit α-amylase by forming aromatic–aromatic interactions (Table 3). NRPET and VEEGNM were suggested to inhibit DPP IV due to the presence of proline in the first four positions and branched chain amino acids at the N-terminal end [112].

#### 5.3.3. Plant Seeds and Nuts

Plant seeds and nuts are excellent sources of proteins, vitamins, minerals and unsaturated fats and is widely recommended as part of a balanced diet. Abundant evidence shows that regular adequate consumption of seeds and nuts impart significant health benefits and protects against several chronic diseases [113]. Peptides identified from various nuts and seeds have also been shown to regulate diabetes by improving glucose metabolism via different mechanisms [114]. Walnut (*Juglans mandshurica)* hydrolysed peptides (WHPs) have produced an α-glucosidase inhibitory rate of 61.73% in in vitro studies [115]. Additionally, administration of WHPs in streptozotocin-induced type 2 diabetic mice resulted in a reduction of fasting glucose level and increase in insulin secretion [115]. The peptide LPLLR, isolated from walnut protein hydrolysates, improved glucose metabolism by inhibiting both α-glucosidase and α-amylase in high glucose-induced insulin-resistant (IR) hepatic HepG2 cells with an inhibition rate of 50.12% and 39.08% respectively. It increased glycogen synthesis and glucose uptake, and decreased gluconeogenesis by activating IRS-1/PI3K/Akt and AMPK signalling pathways [116]. Cationic peptide fractions obtained from flaxseed protein hydrolysate have also demonstrated antidiabetic activity by promoting glucose uptake in L6 myoblast cells [117]. Peptides ACGNLPRMC, ACNLPRMC, and AGCGCEAMFAGA possessing α-amylase inhibitory activity were isolated from basil seeds (*Ocimum basilicum*) using in vitro assays. In silico molecular docking analysis indicated that these peptides bound by interacting with key residues in the active site of the enzyme [118]. An α-amylase inhibitory peptide has also been identified from cumin seeds, an important aromatic spice. Based on in vitro assays, the peptide FFRSKLLSDGAAAAKGALLPQYW inhibited porcine pancreatic α-amylase with an IC_50_ of 0.02 µM [119].

**Table 3:** Antidiabetic action of peptides and protein hydrolysates identified from plant food sources.

Source	Enzyme(s) Used	Substrate	Proteins Hydrolysate	Peptide(s) Identified	Mechanism of Action	IC_50_	Reference
Soy bean				ASCNGVCSPFEMPPCGSSACRC IPVGLVVGYCRHPSG (aglycin)	Enhanced insulin receptor signalling pathway and enhanced glucose uptake in vivo		[85]
Soy bean				VSCNGVCSPFEMPPCGSSACR CIPYGLVVGNCRHPSG (vglycin)	Enhanced insulin signalling by activating the insulin receptor (IR)/Akt signalling pathway		[86,87]
					Restored impaired insulin signalling, glucose tolerance and pancreatic function in vivo		
Soy bean		β-conglycinin		YPFVV (soymorphin-5)	Improved glucose and lipid metabolism via activation of the adiponectin and PPARα system- in vivo		[88]
Soy bean	Pepsin and pancreatin	Germinated soybeans	Protein hydrolysates		Inhibited DPP IV, α-amylase and α-glucosidase in vitro	DPP-IV inhibition- 1.49 mg/mL)	[89]
						α-amylase inhibition- 1.70 mg/mL	
						Maltase and sucrase activities of α-glucosidase 3.73 and 2.90 mg/mL	
Lupin bean				LTFPGSAED	Inhibited DPP IV in situ	207.5 µM	[90]
Soy bean				IAVPTGV		223.2 µM	
Fermented soy bean - Natto		Water soluble extract		KL	Inhibited DPP IV in vitro	41.40 ± 2.68 μg/mL	[96]
				LA		598.02 ± 18.35 μg/mL	
Black bean	Proteinase K, pepsin, trypsin, papain, alcalase, flavourzyme, themolysin, and chymotrypsin		Protein hydrolysates	EGLELLLLLLAG	Inhibited DPP IV in vitro and in silico		[97]
				AKSPLF			
				FEELN			
				TTGGKGGK	Inhibited α-glucosidase in vitro and in silico		
				AKSPLF			
				WEVM	Inhibited α-amylase in vitro and in silico		
Pinto beans	Protamex		Protein hydrolysates	PPHMLP	Inhibited α-amylase in vitro	1.97 mg/mL	[98,99]
				PLPTGAGF		8.96 mg/mL	
				PPHMGGP		14.63 mg/mL	
				PLPLHMLP		18.45 mg/mL	
				LSSLEMGSLGALFVCM		20.56 mg/mL	
Common beans	Pepsin, and pancreatin		Protein hydrolysates		Lowered blood glucose level by inhibiting α-glucosidase		[100]
Bambara bean	Alcalase and thermolysin		Protein hydrolysates		Inhibited DPP IV in vitro	1.73 mg/mL	[101]
Cow pea			Peptides		Activated the insulin signalling pathway in vivo		[102]
Chickpea	Pepsin, pancreatin and bromelain		Protein hydrolysates	PHPATSGGGL	Inhibited DPP IV in vitro	245 μg/mL	[103]
				YVDGSGTPLT			
Oats	Pepsin, trypsin, pancreatin, and alcalase	Globulin		LQAFEPLR	Inhibited DPP IV in vitro	103.5 μM	[106]
				EFLLAGNNK			
Wheat	Debitrase HYW20	Gluten	Gluten hydrolysates	VPL WL	Inhibited DPP IV in vitro		[107]
Quinoa	Pepsin and pancreatin		Protein hydrolysates	GEHGSDGNV	Inhibited DPP IV and α-glucosidase in vitro		[109]
				IQAEGGLT	Inhibited DPP IV and α-glucosidase in vitro		
				DKKYPK	Inhibited α-glucosidase in vitro		
Quinoa	Papain, ficin and bromelain	Seed storage proteins		MAF	Inhibited DPPIV in vitro		[110]
				NMF			
				HPF			
				MCG			
Amaranthus	Alcalase	Albumin and globulin	Protein hydrolysates		Inhibited DPP IV in vitro and in vivo	0.12 ± 0.01 mg/mL	[111]
Amaranthus	Pepsin and pancreatin		Protein hydrolysates	FLISCLL	Inhibited α-amylase and DPP IV in vitro		[112]
				SVFDEELS			
				DFIILE			
				NRPET			
				HVIKPPS			
Walnut	Alcalase, Trypsin, Neutrase, Protamex and Flavourzyme		Protein hydrolysates	LPLLR	Inhibited both α-glucosidase and α-amylase in vitro		[115,116]
Cumin seeds	Protamex		Seed protein hydrolysates	FFRSKLLSDGAAAAKGALLPQYW	Inhibited porcine pancreatic α-amylase in vitro		[119]
Basil seeds	Alcalase		Seed protein hydrolysates	ACGNLPRMC	Inhibited α-amylase activity in vitro		[118]
				ACNLPRMC			
				AGCGCEAMFAGA			

Integrated in silico bioinformatics-based approaches involving proteins from soybean, flaxseed, rapeseed, sunflower, and sesame have identified several potential peptides with DPP IV and ACE inhibitory activity [120]. However, additional in vitro or in vivo experiments would be required to validate these findings. Nonetheless, these approaches highlight the vast library of cryptic peptides that have the potential to inhibit key enzymes in pathways associated with diabetes.

## 6. Structural Characteristics of Antidiabetic Peptides from Food

Numerous studies have demonstrated that bioactive peptides derived from food exhibit physiological functions that could be attributed to their sequence and amino acid composition. The structure and function of the peptide produced is often determined by substrate pre-treatment, enzyme used and hydrolysis conditions. Several of the protein hydrolysates and peptides mentioned here exhibit their antidiabetic activity by inhibiting DPP IV [55,60,63,66,76,90,106]. Evidently, a number of animals, fish and plant proteins could be used as templates for the generation of DPP IV inhibitory peptides. Milk is one of the most extensively studied sources for the generation of peptides. Several milk protein hydrolysates and peptides with antidiabetic activity have been identified from cow, donkey, goat, and camel sources [52,53,54,56]. Other food sources such as egg, meat, pulse, and fish proteins could also potentially contribute antidiabetic peptides [64,72,77]. Interestingly, structure–activity relationship studies have shown that the presence of specific amino acids, as well as their position, could influence DPP IV inhibitory activity. Often, hydrophobic amino acids such as alanine, glycine, isoleucine, leucine, phenylalanine, proline, methionine, tryptophan, and valine are abundantly present in the DPP IV inhibitory peptides. Hydrophilic amino acids such as threonine, histidine, glutamine, serine, lysine, and arginine are also present in many of these peptides [121]. However, the precise role of these hydrophilic amino acids is not well understood. The presence of hydrophobic pockets in the active site of the DPP IV is thought to play an important role in the inhibition of the enzyme by peptides [122]. Moreover, several studies have reported that the presence of a proline residue at the N-terminal region could be a good indicator of DPP IV inhibition. Hatanaka et al. identified 14 different proline-containing dipeptides that could inhibit DPP IV. Interestingly, the position of residues could also determine the activity of a peptide. For instance, the dipeptide Pro-Ile did not inhibit DPP IV activity, while the reverse peptide Ile-Pro competitively inhibited DPP IV. It was also reported that proline-containing dipeptides and tripeptides are also resistant to gastrointestinal digestion [21]. Furthermore, peptides containing proline at the penultimate position was also shown to inhibit DPP IV by substrate type inhibition, where peptides bind to the active site and are subsequently degraded into smaller fragments [57]. Quantitative structure–activity relationship (QSAR) studies have also positively correlated the hydrophobicity of the two amino acids located at the N-terminal end of a peptide with its DPP IV inhibitory potency [57].

The presence of high molecular weight amino acids and aromatic residues such as phenylalanine, tryptophan, tyrosine, and arginine have been observed in peptides that inhibit α-amylase. This is due to the fact that substrate-binding pockets of the α-amylase enzyme has a number of aromatic residues. Hence, apart from interactions involving hydrogen bonds, electrostatic and van der Waals interactions, aromatic–aromatic interactions also play a crucial role in α-amylase inhibitory activity [112,119]. An analysis of 43 α-glucosidase inhibitory peptides suggested that the presence of hydroxyl or basic amino acids at the N-terminal could be responsible for the potency of these peptides. The presence of proline within the peptide and alanine and methionine at the C-terminal could also enhance the inhibitory potential of peptides [123].

## 7. Oral Bioavailability and Stability of Peptide Drugs

Due to their bioactivity, peptides are promising lead molecules for the development of nutraceuticals. Despite their potential, progress is limited due to their poor pharmacokinetics properties. The major challenges faced by peptide drugs include poor oral bioavailability and increased proteolytic instability when compared to small molecule drugs as well as manufacturing costs [124]. However, over the past few decades, new technologies and drug formulations have improved the clinical application of peptide drugs [19]. Oral administration is considered to be the most preferred and convenient way for delivering drugs [125]. Due to poor oral bioavailability, peptide drugs are commonly administered as intravenous, intraperitoneal, or intramuscular injections. However, long-term continuous injection could cause pain and discomfort among patients. Alternate routes are now being sought to deliver peptide drugs safely and effectively [126]. Physical and chemical methods could improve the oral bioavailability and stability of the peptides [127]. This includes pH modulation of intestinal enzymes by adding organic acids such as citric acid and enzyme inhibitors, enteric coating using polyacrylic polymers, and nanoparticles [128,129]. Nanoparticle-coated drugs have shown enhanced bioavailability and distribution [130]. An oral carrier of insulin composed of polysaccharides and nano-silica, which is now in Phase II clinical trials, has been shown to safely lower blood glucose levels [131]. Stability of peptides could also be improved by various chemical approaches such as peptide cyclization and conjugation. Cyclic and conjugated peptide drug candidates for various diseases are in clinical and preclinical trials [132,133]. For example, a PEGylated insulin analog that could be orally delivered is now in Phase II/III clinical trials [134]. The alkylated PEG and permeation enhancer in the insulin analog was shown to increase water solubility, stability, and to enhance absorption.

Despite these advances, bioavailability and stability of peptide drugs is still a major challenge [19]. Nonetheless, a growing number of technological advances in this field is likely to break some of these barriers.

## 8. Future Prospects of Peptide Drugs

Currently, more than 80 peptide drugs are on the market for the treatment of various endocrine, metabolic, and cardiovascular disorders as well as cancer. More than 150 peptides are in clinical trials and nearly 600 peptides are undergoing preclinical studies [19]. The remarkable increase in the number of approvals for peptide-based therapeutics in the last two decades bodes well for an expected acceleration in the near future. Peptide drugs with improved potency and specificity are expected to increase in near future. Additionally, use of conjugated peptide drugs and membrane-penetrating peptides in treating various metabolic syndromes including T2DM will become more common in the coming years. Interest in peptide therapeutics is expected to significantly increase once effective delivery platforms are in place. Furthermore, introduction of novel formulations and chemical methods to stabilize peptide drugs could improve their efficacy. As technology advances, this field is expected to open new horizons in the development of safe and more potent drugs for the treatment of several disorders including diabetes.

## 9. Conclusions

A mounting body of evidence supports the importance of functional foods and food-derived bioactive peptides in managing and treating T2DM. A myriad of bioactive peptides, obtained from various food sources, could be utilized as antidiabetic agents, food supplements or lead compounds in drug discovery. Here, we discuss the potential antidiabetic peptides isolated from a variety of food sources that are capable of inhibiting α amylase, α glucosidase, and DPP IV. Salient structural characteristics of these bioactive peptides have also been summarized, which should assist the design of more potent and specific bioactive peptides. Even though several studies have shown the importance of food-derived bioactive peptides in managing diabetes, their effect on humans is still being actively studied. There still exists a wide gap between scientific studies and commercialization of food-based bioactive peptides. Additional work is required to validate the antidiabetic properties of these peptides through cellular assays and clinical trials for ensuring their efficacy and safety. Much more work needs to be done in the field of peptide therapeutics and delivery systems for improving stability, promoting bioavailability, and enhancing half-life. These studies will eventually help in the commercial production of these peptides as nutraceuticals that could contribute to human health.

## Figures and Tables

**Table 2:** Antidiabetic action of peptides and protein hydrolysates identified from fish.

Source	Enzyme(s) Used	Substrate	Proteins Hydrolysates	Peptide(s) Identified	Mechanism of Action	IC_50_	Reference
Silver carp	Trypsin, neutrase, alcalase, papain, pepsin, and flavourzyme	Dorsal muscle	Muscle protein hydrolysates	LPIIDI	Inhibited DPP IV in vitro	105.44 μM	[76]
				APGPAGP		229.14 μM	
				AGPPGPSG		Nd	
				ALAPSTM		Nd	
Silver carp	Papain, bromelain, alcalase 2.4 L, neutrase, and flavourzyme	Swim bladder protein	Protein hydrolysates	WGDEHIPGSPYH	Inhibited DPP IV in vitro and improved glucose uptake in INS cells	0.35 ± 0.01 mM	[77]
				IAQPQEKAPDPFRH		0.81 ± 0.01 mM	
				IAGPAGPRGPAGPN		1.17 ± 0.04 mM	
				VAPEEHPTL		1.93 ± 0.02 mM	
				YALPHAI		2.27 ± 0.04 mM	
				EPGNPGPAGPA		2.94 ± 0.01 mM	
Tuna	Protease XXIII, orientase 90N	Cooking juice	Cooking juice hydrolysates	PGVGGPLGPIGPCYE	Inhibited DPP IV in vitro	116.1 μM	[78]
				CAYQWQRPVDRIR		78 μM	
				PACGGFWISGRPG		96.4 μM	
Blue whiting	Alcalase and flavourzyme 500	Fish meat	Meat protein hydrolysates		Inhibited DPP IV and mediated insulin and GLP-1 release from BRIN-BD11 and GLUTag cells		[79]
Salmon	Alcalase and Flavourzyme	Skin and trimmings	Protein hydrolysates		Inhibited DPP IV and mediated insulin and GLP-1 release from BRIN-BD11 and GLUTag cells		[80]

Nd—Not determined.
